# Supplementary material for: Content-rich biological network constructed by mining PubMed abstracts
Source: BMC Bioinformatics. 2004 Oct 8;5:147. doi: 10.1186/1471-2105-5-147 (PMC528731; doi:10.1186/1471-2105-5-147)
Supplement: Additional File 5 — The original Chilibot query results of the term "long-term potentiation (LTP)" and 22 other terms, limiting the latest references analyzed to the years 1990, 1995, 2000, and 2004. [file 1471-2105-5-147-S5.bz2 › chilibotAdditionalFile5/ltp1995/html/PKA_TAU.html]

 


 **PKA** and **TAU** 
  
Found 28 abstracts in PubMed,  **28 abstracts were retrieved and analyzed**.  


---

 Search Google  |
 PDF files only 
|  EDU domain only 

---

**Interactive relationship** (e.g. stimulation, inhibition, etc)

- **PKA**  phosphorylated  **tau**  and reduced its electrophoretic mobility, phosphorylation towards the C terminus of  **tau**  having a major influence on this property.  Ref: 8261023 DementiaDementia, 1991
- Interestingly, the actions of different kinases on  **TAU**  are not independent, but may depend on the order in which they work on  **TAU**  ; i.e., prior phosphorylation by  **PKA**  partially inhibits the action of PK40erk.  Ref: 8166686 Biochem Biophys Res Commun, 1994
- In extremely cases this advantage could be significant, and the calculated increase in time required for 99.9% coupling substrates 6  **pKa**  units less reactive than the standard ones reaches 1 00  **tau**  1 2 units for classic active esters, but only 631  **tau**  1 2 units for reaction involving superactive esters.  Ref: 8005755 Int J Pept Protein Res, 1994
- Addition of 250 U ml of protein kinase A  **PKA** , 1 mM ATP, and 5 mM MgCl2 in the bath solution 150 mM Na glutamate increased the single channel  **tau**  1 from 3.26 0.15 to 7.38 0.38 and Po from 0.41 0.06 to 0.72 0.07 n = 6 without altering conductance.  Ref: 8048548 Am J Physiol, 1994
- A recently described protein kinase, PK40erk, 1 a member of the ERK family of kinases, can produce in vitro many of the properties of Alzheimer like hyperphosphorylated  **TAU** . cAMP dependent protein kinase A  **PKA**  phosphorylates  **TAU**  to a lesser extent.  Ref: 8166686 Biochem Biophys Res Commun, 1994
- Although the phosphorylated forms of  **tau**  generated by  **PKA**  are not identical to those of PHF  **tau** ,  **PKA**  may be involved in the generation of PHF  **tau**  in Alzheimer s disease via phosphorylation of additional, as yet unidentified, sites on  **tau** .  Ref: 8261023 DementiaDementia, 1994
- Thus, prior phosphorylation by  **PKA** , a non proline directed protein kinase, regulates the conformation of the protein substrate  **Tau**  so as to make some sites very much less accessible to phosphorylation by the proline directed kinase, PK40erk2.  Ref: 7488031 Biochem Biophys Res Commun, 1995
- Here we show that the sites of phosphorylation by four kinases  **PKA** , PKC, CK and CaMK all lie in the C terminal microtubule binding half of  **tau** , but only the phosphorylation by CaM kinase shows the pronounced shift in electrophoretic mobility characteristic for  **tau**  from Alzheimer neurofibrillary tangles.  Ref: 2120043 EMBO J, 1990

**Parallel relationship** (e.g. studied together, co-existance, homology, etc.)

- Substitution of serine396 phosphorylated in PHF  **tau**  or serine416 phosphorylated by calcium calmodulin kinase II by alanine demonstrated that these are not major sites for  **PKA**  phosphorylation.  Ref: 8261023 DementiaDementia, 1990
- Fenoldopam had an affinity  **pKA**  of 5.84 0.04 and efficacy  **tau**  of 0.57 0.04.  Ref: 1361397 Br J Pharmacol, 1992
- We have investigated the effects of cyclic AMP dependent protein kinase  **PKA**  on recombinant human  **tau**  isoforms and two recombinant  **tau**  fragments.  Ref: 8261023 DementiaDementia, 1992
- ... 5 HT had a  **pKA**  of 6.65 0.12 and  **tau**  of 2.66 0.41.  Ref: 1361397 Br J Pharmacol, 1992
- Hyperphosphorylation of human  **TAU**  by brain kinase PK40erk beyond phosphorylation by cAMP dependent  **PKA**  relation to Alzheimer s disease.  Ref: 8166686 Biochem Biophys Res Commun, 1994
- 4 still present during stimulation or inhibition of adenosine cyclic monophosphate dependent protein kinase  **PKA**  by 10 microM forskolin or 1 microM H 89, respectively SS and PR  **tau**  values were abbreviated and prolonged, respectively.  Ref: 7864197 Am J Physiol, 1995
